# Fear of the Unknown: Does Fear of Terrorism Differ From Fear of Contracting COVID-19?

**DOI:** 10.3389/fpsyg.2021.660777

**Published:** 2021-06-11

**Authors:** Mally Shechory Bitton, Avital Laufer

**Affiliations:** ^1^Department of Criminology, Ariel University, Ariel, Israel; ^2^Netanya Academic College, Netanya, Israel

**Keywords:** COVID-19, terrorism, fear, cope, resilience

## Abstract

The aim of the study was to explore whether living under constant security threat would result in better coping and higher resilience when exposed to an unknown threat such as the COVID-19 pandemic. Thus, fear of COVID-19 and fear of terrorism as well as the associations with coping strategies and resilience were examined among Israelis living in conflict zones as well as Israelis living in the center, where exposure to security incidents is rare. Six hundred and fifteen Israeli adults (260 men and 356 women) were interviewed via the internet while Israel was under mandatory first lockdown. Fear of COVID-19 was found to be higher than fear of terrorism among both groups. those living in the conflict zones and those living in the central Israel. In contradiction to our assumption, we found that those who were living in a conflict zone did not exhibit higher levels of resilience and did not cope better when exposed to a new threat—even though they may be more skilled at handling prolonged exposure to a threat such as terrorism. A regression analysis indicated that the best predictor of both fear of COVID-19 and of terrorism is financial concerns—more than geographical area.

## Introduction

Since the beginning of the twenty first century, many countries around the globe have had to cope with different epidemics and viral outbreaks (for review see: Brooks et al., 2020). COVID-19 is considered the largest pandemic in the twenty first century (Singhal, 2020). The global impact of COVID-19 and the public health threat it represents are the most serious seen in a respiratory virus since the 1918 H1N1 influenza pandemic (Ferguson et al., 2020). Despite the ongoing development of vaccines, the pandemic is not yet over. Many countries have been experiencing multiple waves of COVID-19 outbreaks.

A powerful effect of exposure to COVID-19, particularly in the first stage, was the sense of fear due to the uncertain nature of the threat. This following intensive reports in all forms of media concerning high rates of morbidity and mortality, side by side with the lack of knowledge and concern of an insufficient medical response, as well as threats that the healthcare system would collapse. Public anxieties and concerns were and continue to be high, along with an apparent wave of fear and worry in society (Lin, 2020). Research has shown that COVID-19 is perceived as a new, unknown, and out-of-control hazard and source of intense fear for the entire global population (Ahorsu et al., 2020; Rajkumar, 2020). People have reported fear of infection, death, loss of a family member, and contact with people who may be infected (Brooks et al., 2020; Fardin, 2020; Mertens et al., 2020), as well as career and financial loss (Trzebiński et al., 2020). These reports may seem quite similar to the effect of terrorism and security threats. Hence, the current study is the first, as far as we know, to examine and compare the fear associated with security threats and fear of COVID-19. The main aim of the study was to examine whether Israelis habituated to living in conflict areas and being exposed to security threats experience a new type of threat, in the form of COVID-19, as more frightening than the familiar threat of terrorism incidents.

### Threat of Terrorism vs. the Threat of COVID-19

Terrorism and COVID-19 share common elements. They affect the social fabric of life by creating a sense of fear and interfering with normal daily life routines. Both have psychological, physical, and financial effects. Similar to the effects described above due to exposure to COVID-19, the impact of terrorism is not restricted to the individual, but rather extends to entire communities (Perry and Alvi, 2011).

High levels of individual and public fear and distress were found following exposure to security and terrorism events (e.g., Haner et al., 2019). Fear and worry about terrorism attacks have been found to prompt several behavioral adjustments to individuals' daily life in order to minimize the risk of falling victim to a future attack, even if they themselves had not been directly victimized (Eisenman et al., 2009; Denovan et al., 2017).

At the same time, the source of each of these threats is quite different. Terrorism is a geopolitical threat, man-made acts that are uniquely motivated by ideology aimed at achieving political objectives. It is designed to harm random victims, cause damage and death, and plant seeds of fear and chaos far beyond its immediate victims and among a wider public (Perry and Alvi, 2011; Romanov et al., 2012). Thus, fear of terrorism may be linked to geographical location (Benzion et al., 2009; Besser and Neria, 2012).

Another difference is the visibility of the enemy. While in the case of terrorism the enemy is normally known, when dealing with a pandemic it is almost impossible to pinpoint a person or body agent that intentionally caused the spread of the virus. As such, COVID-19 is an invisible health threat as opposed to a visible enemy.

Regardless of the dissimilarities and discrepancies of these stressors, it appears that high levels of individual and public fear and distress were found following exposure to security and terror events (e.g., Haner et al., 2019) as well as fear of getting infected from COVID-19 (Lin, 2020). Various studies indicate processes of habituation following lengthy exposure to threatening and stressful situations (Bensimon, 2012; Stein et al., 2018; Laufer and Shechory Bitton, 2020). Studies that examined populations living in conflict areas, including Israel, and exposed to lengthy security threats, reported a lower sense of fear and distress than situations of exposure to a one-time event (Itzhaky et al., 2017; Shechory Bitton and Laufer, 2018; Shechory Bitton and Silawi, 2019). Fear and worry about terror attacks have been found to prompt several behavioral adaptations in individuals' daily lives in order to minimize the risk of falling victim to a future attack, even if they themselves had not been directly victimized (Eisenman et al., 2009; Denovan et al., 2017).

### The Situation in Israel

Israelis, who have considerable experience dealing with life under a constant security threat, are now faced with a new reality in which they are required to deal with an unfamiliar situation, dealing with an epidemic that is a threat to their health and to the health of their family, when uncertainty and lack of knowledge concerning the virus and its effects were at their height (Shechory Bitton and Laufer, 2021). Although there have been several global epidemics in recent decades, Israel was hardly affected by them. In fact, it could be argued that the last epidemic with which Israel had to cope was polio in the 1950s (Swartz, 2008). Since then, Israeli society has mostly had to contend with terrorism and the threat of terrorism has occupied a central place in the Israeli collective experience (Herzenstein et al., 2015). However, exposure to COVID-19 has affected Israeli society (Shrira et al., 2020).

Accustomed as it is to coping with terrorism and security threats, Israel is now facing a health threat caused by an invisible enemy. As such, this unique situation in Israel enables us to examine whether the development of resilience in the context of terrorism, following prolonged exposure, also affects resilience in the context of dealing with a pandemic. Thus, we examined the differences between two kinds of fear: of terrorism and of the pandemic, and we also examined whether fear of the pandemic and coping with the pandemic differ between those residing in areas of Israel confronted with a daily threat of terrorism and war and those residing in central Israel who are not exposed to fear of terrorism and war on a daily basis.

In contrast to the new COVID-19 threat extending to Israel's entire population, coping with terror events in Israel is geographically dependent. While inhabitants of border areas must deal with belligerent incidents on a daily basis, in central Israel there is almost no exposure to such events. Over the years, those living in areas of conflict have been exposed to attacks, both directly as well as indirectly through the injury or death of friends (Laufer et al., 2009; Shechory Bitton, 2013).

### Resilience and Coping Strategies

Resilience and coping constitute a major component of people's ability to cope with stressful events (Connor and Davidson, 2003; Besser et al., 2015; Hu et al., 2015). A growing body of research shows that resilience, indicated either by a low rate of post-traumatic stress symptoms or distress, is the most common reaction to traumatic events (e.g., Bonanno et al., 2012; Stein et al., 2018). Resilience can be defined as the ability to cope successfully with stressful and traumatic events and retain a sense of equilibrium (Bonanno, 2004; Straud et al., 2018).

In fact, resilience in the face of adversity is the capacity to move ahead under adverse circumstances, a human response that leads to better health, both mentally and physically. Resilient people were found to enjoy better physical and mental health, lower levels of depression, anxiety, sleep disorders, and PTS, compared to people who are less resilient (Hu et al., 2015; Straud et al., 2018; Finklestein et al., 2020).

In the current study, we follow Connor and Davidson (2003) definition of resilience as a personal characteristic that embodies the personal qualities as well as an individual's past life experiences and current life circumstances enabling one to thrive in the face of adversity.

Resilience has also been associated with coping strategies in the context of various adverse events (Reich et al., 2010). Lazarus and Folkman (1984, 1991) suggested two major forms of coping: problem-focused (dealing with stress sources and taking proactive steps to change them) and emotion-focused (serving to reduce the emotional stress resulting from such situations) (see also Folkman, 2013). Mostly, greater use of emotion-focused coping is highly correlated with high levels of psychological distress (e.g., Carver and Scheier, 1993; Gilbar et al., 2010; Rodrigues and Renshaw, 2010). In contrast, use of problem-focused strategies has been negatively correlated with distress and indicates good mental health (Taft et al., 2007; Gilbar et al., 2010) and higher levels of resilience (Li and Nishikawa, 2012; Thompson et al., 2018).

Several studies conducted during the ongoing coronavirus pandemic, found that resilient people and individuals who use positive, active, or problem-focused coping, worry less and have fewer symptoms of anxiety and depression than people who do not (Barzilay et al., 2020; Haven et al., 2020; Yu et al., 2020). However, the findings are not unequivocal. Other studies have identified a “coping-panic cycle” in which the more one uses coping (whether emotion-focused or problem-focused), the more one experiences distress during this pandemic (Huang et al., 2020; Man et al., 2020).

When examining the role of coping with exposure to terrorism and security threats, findings show that both coping strategies can be positively correlated with pathogenic (e.g., distress and fear, PTS symptoms) as well as with salutogenic factors (e.g., Shechory Bitton and Laufer, 2017). Some findings even emphasize the importance of emotion-focused strategies *in situ*ations perceived as uncontrollable or in the absence of a viable solution (e.g., exposure to terrorism and security threats) (May et al., 2011; Besser and Neria, 2012; Braun-Lewensohn and Mosseri Rubin, 2014).

According to the goodness of fit hypothesis, the effectiveness of different coping strategies depends on the appraised controllability of the event. Problem-focused strategies are proposed to be adaptive *in situ*ations perceived as controllable and maladaptive *in situ*ations perceived as uncontrollable. In contrast, high levels of emotion-focused coping are proposed to have positive effects on adaptation in uncontrollable situations (Conway and Terry, 1992). In these cases, it may even be better to use emotion-focused coping, since this strategy may reduce the negative psychological effects of the event without confronting it directly (Zeidner, 2006; Shechory Bitton and Cohen-Louck, 2021).

### The Current Study

The current study was conducted during the peak of the first COVID-19 lockdown in Israel, when uncertainty and lack of knowledge concerning the virus and its effects were at their height. On March 17, 2020, the Israeli government officially imposed a lockdown. At the time of data collection (March 30 to April 8, 2020), Israel's borders were closed and the government had instructed residents to remain at home while imposing limitations on the public and private sectors. Many people were forced to stop working, with no knowledge of when and even whether they would be returning to their jobs. The stay-at-home order was implemented rigorously, resulting in increasing unemployment in many areas of the economy, with the national unemployment rate rising from 3.4 to 27% in April 2020. Thus, Israeli society was confronted with a new and unfamiliar threat.

The main aim of the study was to examine whether Israelis habituated to living in conflict areas and exposed to security threats experience a new threat type, in the form of COVID-19, as more frightening than the familiar threat of terrorism incidents. That is, whether a previous habituation process of living in a conflict zone, will be manifested in a higher level of resilience and coping abilities with the new threat of COVID-19. As such, the study aimed to examine the levels of fear, resilience, and coping ability of two Israeli groups, those living in conflict areas who were previously found to have higher levels of habituation to fear due to terrorism (e.g., Stein et al., 2018) and those living in central Israel, where exposure to security incidents is rare. Thus, fear of COVID-19 and fear of terrorism as well as the associations with coping strategies and resilience were examined.

Previous findings suggested that gender differences are a dominant indicator of the response to stressful situations, with higher symptomatology among women compared to men (e.g., Laufer et al., 2019; Shechory Bitton and Cohen-Louck, 2021). Thus, gender differences were also examined.

Based on the literature review we hypothesized that those who were living in a conflict zone would exhibit lower levels of fears and higher levels of resilience and would cope better when exposed to the new pandemic threat. In addition, we hypothesized that lower level of resiliency and maladaptive coping strategy (emotion-focused coping more than problem-focused coping) would predict higher levels of fear of both threats.

## Method

### Participants

The participants in this study numbered 615 individuals: 260 males (42.3%) and 355 females (57.7%). Two hundred and fifty-one of the participants were living in conflict areas (40.8%) and 364 in central Israel (59.2%), with no gender differences by area (Z = 1.85, *p* = 0.065). The mean age of the participants was 47.54 (SD = 14.50, range 19–88), with no difference by area [*F*_(1, 500)_ = 1.61, *p* = 0.206, η^2^ = 0.003], by gender [*F*_(1, 500)_ = 1.81, *p* = 0.179, η^2^ = 0.004], or by the interaction of area with gender [*F*_(1, 500)_ = 0.58, *p* = 0.445, η^2^ = 0.001].

Most of the sample from both groups were married and parents of children (75.4 and 84.7%, respectively). Overall, the number of children per family ranged from 0 to 11 (M = 2.61, SD = 1.62). Significant differences were found by area [*F*_(1, 573)_ = 16.56, *p* < 0.001, η^2^ = 0.003], but no difference by gender [*F*_(1, 573)_ = 0.27, *p* = 0.601, η^2^ = 0.001] or by the interaction of area with gender [*F*_(1, 573)_ = 1.32, *p* = 0.251, η^2^ = 0.002]. Families in conflict areas had more children (M = 2.92, SD = 1.72) than families in central Israel (M = 2.39, SD = 1.51).

Most of the participants (*N* = 401, 65.2%) had an academic education, to a greater extent in central Israel (*N* = 257, 70.6%) than in conflict areas (*N* = 144, 57.4%) (Z = 3.39, *p* < 0.001), and to a greater extent among females (*N* = 252, 71.0%) than males (*N* = 149, 57.3%) (Z = 3.52, *p* < 0.001). A quarter of the participants (*N* = 155, 25.2%) continued to commute to work at the time, while all others worked from home (*N* = 187, 30.4%) or did not work (*N* = 273, 44.4%). Continuing to go to work did not differ by area (Z = 1.27, *p* = 0.203), but was higher among males (*N* = 92, 35.4%) than females (*N* = 63, 17.7%) (Z = 4.98, *p* < 0.001). Most participants who were living in central Israel (*N* = 201, 62.8%) were secular, while most participants in conflict areas (*N* = 113, 56.8%) were non-secular (Z = 4.37, *p* < 0.001). No gender differences were found by religiosity (Z = 1.56, *p* = 0.119). In addition, only 8 participants (1.3%) reported that they had direct contact with someone who had become infected with COVID-19 at the time of collecting the data.

No differences were found between the groups in their response to the question: How concerned are you about your financial situation due to the COVID-19 crisis? Concern about one's financial situation due to COVID-19 differed only by gender [*F*_(1, 611)_ = 6.64, *p* = 0.010, η^2^ = 0.011], with females reporting greater concern (*M* = 2.85, *SE* = 0.07) than males (*M* = 2.58, *SE* = 0.08).

#### Measurements

##### Personal Data

In order to record demographic variables, respondents were asked to provide information about their gender, age, marital status, level of education, religiosity, and place of residence. In addition, questions were asked focusing on the lockdown due to COVID-19: Did you/ a family member contract COVID-19? (yes or no); Did you continue working during the lockdown? (yes or no). Also, one question was addressed the financial situation: How concerned are you about your financial situation due to COVID-19? (on a 5-point scale: 1 = not at all to 5 = very much).

##### Fear of Terrorism and Fear of Contracting COVID-19

The respondents were asked to answer 5 questions that examined their level of fear of being attacked by terrorists or of contracting the COVID-19 virus, as well as their level of fear that their family members would be affected, on a 5-point scale (1 = not at all to 5 = very much): (1) To what degree are you afraid that your health will be affected due to contracting COVID-19?; (2) To what degree are you afraid that your family members' health will be affected due to contracting COVID-19?; (3) To what degree are you afraid at present of being hurt in a terrorist incident?; (4) To what degree are you afraid at present that someone in your family will be hurt in a terrorist incident? In addition, the respondents were asked to answer the question: Compared to your fear of contracting COVID-19, how afraid are you at present of being hurt in a terrorist incident? The response options were: 1. More afraid of contracting the COVID-19 virus; 2. More afraid of being hurt in a terrorist incident; 3. Equally afraid; 4. Not afraid of being hurt by either of them.

*Coping strategies* were measured using the COPE scales (Carver et al., 1989). The COPE scales examine two major coping strategies: problem-focused (15 items) and emotion-focused (15 items). Participants were asked to rate the extent to which they used each coping option to deal with stressful situations (e.g., exposure to COVID-19), on a 4-point scale (0 = not at all; 3 = a great deal) (data were transformed into a 1–4 scale). Higher mean scores on each dimension indicate more frequent use of that coping style. Cronbach's alpha was 0.83 for problem-focused and 0.68 for emotion-focused coping. The scale has been used extensively in Hebrew (e.g., Shechory Bitton, 2014).

##### Coping Strategies

Coping strategies were examined via use of the Coping Orientation to Problems Experienced scale (COPE scale; Carver et al., 1989). This scale assesses problem-focused strategies (15 items) and emotion-focused strategies (15 items). Participants were asked to rate the extent to which they used each coping option to deal with stressful situations on a scale ranging from 0 (not at all) to 4 (a great deal). Data were transformed into a 1–4 scale. The scale has been used extensively in Hebrew, showing good predictive validity and internal consistency (e.g., Shechory Bitton, 2014). In the current study, Cronbach's α for problem-focused coping was 0.82, and 0.68 for emotion-focused coping.

*Resilience* was measured using the Connor-Davidson Resilience Scale (CD-RISC; Connor and Davidson, 2003). It consists of 25 statements (e.g., able to adapt when changes occur; have close and secure relationships; belief that one can deal with whatever comes; and have control of one's life). Each statement is rated by respondents for their extent of agreement with it (0 = not at all to 4 = true nearly all the time). Total CD-RISC scores representative of resilience were utilized for this study (α = 0.89). The scale has been used in Hebrew (Finklestein et al., 2020).

#### Procedure

The study was a cross-sectional survey study, based on respondent self-reports through an online survey. Considering the feasibility of electronic questionnaires, a professional online questionnaire powered through an online survey platform was used to complete the paperless survey. Inclusion criteria were: adult Israeli citizens (over 18 years old), Jewish, living in a conflict area (southern or northern Israeli border, or the West Bank—Judea and Samaria), or living in central Israel (mainly the Tel-Aviv district). Participants were recruited over 8 days between March 31 and April 8, 2020. As mentioned in the introduction, the study was conducted during the peak of the first COVID-19 lockdown in Israel, when uncertainty and lack of knowledge concerning the virus and its effects were at their height. At that time, the Israeli government had issued a directive for residents to isolate themselves at home and minimize face-to-face interactions. All respondents provided informed consent. The questionnaire stated that participation is anonymous and confidential. The participants were informed that their answers would serve only for research purposes and that they could stop answering at any point. Then, they were required to complete the questionnaires anonymously. The raw data was then transferred into a database. The study was approved according to the ethical standards of the Institutional Review Board (IRB) of Ariel University.

#### Data Analysis

Data were analyzed with SPSS version 27. Background variables were described with means and standard deviations, as well as frequencies and percentages. The background variables were compared by geographical area and gender using analyses of variance and Chi-squares. The research variables were described with means and standard deviations, and compared by geographical area and gender using analyses of variance. Significant interactions were interpreted with estimated marginal means, using Bonferroni adjustments for multiple comparisons.

Differences between fear of COVID-19 and of terrorism, and between fear for oneself vs. one's family, were calculated with a 2 × 2 × 2 × 2 repeated measures analysis of variance (theme of fear—COVID-19/terrorism, subject of fear—self/family, geographical area—conflict area/central Israel, and gender—male/female). Theme of fear and subject of fear served as within subjects variables, and hence the repeated measures analysis of variance. Geographical area and gender served as between subjects variables. Significant interactions were interpreted with estimated marginal means, using Bonferroni adjustments for multiple comparisons. Pearson correlations were calculated among the research variables. Multiple hierarchical regressions were calculated for fear of COVID-19 and terrorism, using background variables and financial concerns in the first step, and coping strategies and resilience in the second.

## Results

### Fear of Terrorism and Fear of COVID-19

First, a total 2 × 2 × 2 × 2 repeated measures analysis of variance was calculated (type of fear—COVID-19/terrorism, subject of fear—self/family, geographical area—conflict area/central Israel, gender—male/female). Results showed that COVID-19 related fear was higher than terrorism related fear, that fear for family was higher than fear for oneself, that women showed higher levels of fear than men, and that overall fear was higher in conflict areas than in central Israel. That is, a significant difference was found for type of fear [*F*_(1, 611)_ = 352.07, *p* < 0.001, η^2^ = 0.366], with COVID-19 related fear (M = 3.04, SE = 0.04) higher than terrorism-related fear (M = 2.15, SE = 0.05). Another significant difference was found for the subject of fear [*F*_(1, 611)_ = 567.58, *p* < 0.001, η^2^ = 0.482], with fear for family (M = 2.92, SE = 0.04) higher than fear for oneself (M = 2.27, SE = 0.04). A small significant effect was found for gender [*F*_(1, 611)_ = 9.45, *p* = 0.002, η^2^ = 0.015], with women (M = 2.71, SE = 0.05) showing more fear overall than men (M = 2.48, SE = 0.06). In addition, a small significant effect was found for geographical area [*F*_(1, 611)_ = 4.62, *p* = 0.032, η^2^ = 0.008], with participants from conflict areas (M = 2.68, SE = 0.06) showing higher fear overall than participants from central Israel (M = 2.51, SE = 0.05).

Further analyses for fear of COVID-19 (Table 1) revealed only one difference, with women showing higher fear for family than men. In other words, fear of COVID-19 with regard to oneself did not differ by geographical area, gender, or their interaction. Fear of COVID-19 with regard to one's family differed by gender, with women showing higher fear (M = 3.56, SE = 0.06) than men (M = 3.36, SE = 0.07), and did not differ by geographical area or by its interaction with gender.

**Table 1:** Distribution of the fear variables by area and gender (*N* = 615).

	**Conflict area**			**Central Israel**			**Difference**		
	**Male (*n =* 95)**	**Female (*n =* 156)**	**Total**	**Male (*n =* 165)**	**Female (*n =* 199)**	**Total**	**Area**	**Gender**	**Area × gender**
Fear for self—COVID-19	2.64 (1.15)	2.58 (1.17)	2.61 (1.16)	2.54 (1.14)	2.71 (1.11)	2.63 (1.13)	*F*_(1, 611)_ = 0.02 (*p* = 0.893) (η^2^ = 0.001)	*F*_(1, 611)_ = 0.31 (*p* = 0.575) (η^2^ = 0.001)	*F*_(1, 611)_ = 1.38 (*p* = 0.240) (η^2^ = 0.002)
Fear for family—COVID-19	3.47 (1.08)	3.58 (1.07)	3.53 (1.08)	3.26 (1.15)	3.53 (1.12)	3.41 (1.14)	*F*_(1, 611)_ = 2.03 (*p* = 0.155) (η^2^ = 0.003)	*F*_(1, 611)_ = 4.24 **(*****p=*** **0.004)** (η^2^ = 0.007)	*F*_(1, 611)_ = 0.83 (*p* = 0.363) (η^2^ = 0.001)
Fear for self—terror	1.88 (1.02)	2.15 (1.17)	2.05 (1.12)	1.52 (0.87)	2.15 (1.12)	1.86 (1.06)	*F*_(1, 611)_ = 4.19 (***p=*** **0.041**) (η^2^ = 0.007)	*F*_(1, 611)_ = 25.29 (***p<*** **0.001**) (η^2^ = 0.040)	*F*_(1, 611)_ = 4.11 (***p=*** **0.043**) (η^2^ = 0.007)
Fear for family—terror	2.55 (1.31)	2.58 (1.34)	2.57 (1.33)	1.96 (1.14)	2.44 (1.25)	2.22 (1.22)	*F*_(1, 611)_ = 11.86 (***p<*** **0.001**) (η^2^ = 0.019)	*F*_(1, 611)_ = 5.73 **(*****p=*** **0.017**) (η^2^ = 0.009)	*F*_(1, 611)_ = 4.47 (***p=*** **0.035**) (η^2^ = 0.007)

*Bold values indicated difference are significant*.

Fear of terrorism regarding oneself was higher in conflict areas and among women, than in central Israel and among men (respectively). That is to say, fear of terrorism with regard to oneself differed by geographical area, with participants from conflict areas (M = 2.01, SE = 0.07) showing more fear than participants from central Israel (M = 1.83, SE = 0.06). It was also higher among women (M = 2.15, SE = 0.06) than men (M = 1.70, SE = 0.07). Analysis of the significant interaction for fear of terrorism regarding oneself revealed that women from both geographical areas had the highest means for fear of terrorism with regard to oneself, whereas men in central Israel had the lowest mean score. More specifically, women reported higher fear of terrorism with regard to themselves than did men in central Israel (*p* < 0.001), but no gender difference was found in conflict areas (*p* = 0.054). Further, men in conflict areas reported higher fear than in central Israel (*p* = 0.008), but no area difference was found among women (*p* = 0.988).

Quite similarly, fear of terrorism with regard to one's family was higher in conflict areas and among women, than in central Israel and among men (respectively). That is, participants from conflict areas (M = 2.56, SE = 0.08) showed higher fear than participants from central Israel (M = 2.20, SE = 0.07). In addition, scores were higher among women (M = 2.51, SE = 0.07) than men (M = 2.26, SE = 0.08). Analysis of the significant interaction for fear of terrorism regarding the family revealed that participants from conflict areas and women from central Israel had higher means for fear of terrorism with regard to their family, than did men in central Israel. More specifically, women reported higher fear of terrorism with regard to their family than did men in central Israel (*p* < 0.001), but no gender difference was found in conflict areas (*p* = 0.856). Further, men in conflict areas reported higher fear than in central Israel (*p* < 0.001), but no area difference was found among women (*p* = 0.298).

Comparing fear of COVID-19 to fear of terrorism with regard to oneself, showed that women were more concerned of terrorism than men, and that fear of terrorism was higher in conflict areas than in central Israel, but no differences were found for fear of COVID-19. That is, two significant interactions were found: fear by gender [*F*_(1, 611)_ = 16.04, *p* < 0.001, η^2^ = 0.026] and fear by geographical area [*F*_(1, 611)_ = 4.49, *p* = 0.034, η^2^ = 0.007]. Interpretation of the first interaction with gender revealed that women were more concerned of terrorism than were men [M = 2.14, SE = 0.06 vs. M = 1.69, SE = 0.07, *F*_(1, 611)_ = 26.32, *p* < 0.001, η^2^ = 0.041], while no significant gender difference was found for fear of COVID-19 [women: M = 2.64, SE = 0.06 vs. men: M = 2.60, SE = 0.07, *F*_(1, 611)_ = 0.23, *p* = 0.633, η^2^ = 0.001].

Interpretation of the second interaction with geographical area revealed that fear of terrorism was higher in conflict areas than in central Israel [M = 2.01, SE = 0.07 vs. M = 1.82, SE = 0.05, *F*_(1, 611)_ = 5.18, *p* = 0.023, η^2^ = 0.008], while no significant area difference was found for fear of COVID-19 [conflict area: M = 2.61, SE = 0.07 vs. central Israel: M = 2.63, SE = 0.06, *F*_(1, 611)_ = 0.02, *p* = 0.881, η^2^ = 0.001]. Further, the discrepancy between the two types of fear, with fear of COVID-19 being higher, was greater in central Israel (η^2^ = 0.212) than in conflict areas (η^2^ = 0.088).

Comparing fear of COVID-19 and fear of terrorism with regard to one's family, showed that fear of terrorism was higher in conflict areas than in central Israel, but no differences were found for fear of COVID-19. That is, no significant interaction was found for fear by gender [*F*_(1, 611)_ = 0.47, *p* = 0.495, η^2^ = 0.001], but a significant interaction was found for fear by geographical area [*F*_(1, 611)_ = 5.10, *p* = 0.024, η^2^ = 0.008]. Its interpretation revealed that fear of terrorism with regard to one's family was higher in conflict areas than in central Israel [M = 2.56, SE = 0.08 vs. M = 2.20, SE = 0.07, *F*_(1, 611)_ = 11.86, *p* = 0.001, η^2^ = 0.019], while no significant area difference was found for fear of COVID-19 with regard to one's family [conflict area: M = 3.52, SE = 0.07 vs. central Israel: M = 3.40, SE = 0.06, *F*_(1, 611)_ = 1.63, *p* = 0.203, η^2^ = 0.003]. Further, the discrepancy between the two types of fear with regard to one's family, with fear of COVID-19 being higher, was greater in central Israel (η^2^ = 0.340) than in conflict areas (η^2^ = 0.177).

Table 2 presents group differences in coping and resilience by area and gender.

**Table 2:** Distribution of coping and resilience by area and gender (*N* = 615).

	**Central Israel**		**Conflict Area**		**Difference**		
	**Male**	**Female**	**Male**	**Female**	**Area**	**Gender**	**Area × gender**
	***M* (*SD*)**	***M* (*SD*)**	***M* (*SD*)**	***M* (*SD*)**			
Coping—problem- focused (0–3)	1.14 (0.55)	1.50 (0.52)	1.22 (0.57)	1.38 (0.53)	*F*_(1, 611)_ = 0.32 (*p* = 0.574) (η^2^ = 0.001)	*F*_(1, 611)_ = 33.13 **(*****p<*** **0.001**) (η^2^ = 0.051)	*F*_(1, 611)_ = 4.79 **(*****p=*** **0.029**) (η^2^ = 0.008)
Coping—emotion- focused (0–3)	0.92 (0.37)	1.10 (0.36)	0.87 (0.35)	1.09 (0.34)	*F*_(1, 611)_ = 1.45 (*p* = 0.229) (η^2^ = 0.002)	*F*_(1, 611)_ = 43.75 (***p<*** **0.001**) (η^2^ = 0.067)	*F*_(1, 611)_ = 0.60 (*p* = 0.438) (η^2^ = 0.001)
Resilience—total score (0–100)	68.11 (13.69)	65.95 (12.04)	70.48 (12.87)	67.36 (13.76)	*F*_(1, 607)_ = 2.97 (*p* = 0.085) (η^2^ = 0.005)	*F*_(1, 607)_ = 5.79 **(*****p=*** **0.016**) (η^2^ = 0.009)	*F*_(1, 607)_ = 0.19 (*p* = 0.663) (η^2^ = 0.001)

*Bold values indicated difference are significant*.

As evident from Table 2, no differences were found between the respondents in coping strategies and resilience by place of residence. Nevertheless, differences related to the respondent's gender were found. Problem-focused coping was higher among women (M = 1.45, SD = 0.053) than among men (M = 1.17, SD = 0.56). A significant interaction showed that this gender-based difference was greater in central Israel [*F*_(1, 611)_ = 39.88, *p* < 0.001, η^2^ = 0.061] than in conflict areas [*F*_(1, 611)_ = 5.27, *p* = 0.022, η^2^ = 0.009]. Similar gender differences were found for emotion-focused coping, with women (M = 1.09, SD = 0.35) having higher scores than men (M = 0.90, SD = 0.36). In addition, a significant gender-based difference was found in the resilience variable, with men (M = 68.98, SD = 13.41) scoring higher than women (M = 66.57, SD = 12.83).

### Pearson Correlations and Multiple Hierarchical Regressions

Means, standard deviations, and Pearson correlations among the research variables are presented in Table 3. Results show that among all types of fear and concern, fear for one's family regarding COVID-19 was highest and fear for oneself regarding terrorism was lowest. Significant relationships were found between the research variables. All types of fear and concern were positively interrelated, and in most cases were positively related with both types of coping strategies. Resilience was negatively and weakly associated with fear for oneself regarding COVID-19, and was positively associated with problem-focused coping.

**Table 3:** Means, standard deviations, and intercorrelations for the research variables (*N* = 615).

	***M* (*SD*)**	**2**.	**3**.	**4**.	**5**.	**6**.	**7**.	**8**.
1. Fear for one's self—COVID-19 (1–5)	2.62 (1.14)	0.66^***^	0.39^***^	0.36^***^	0.37^***^	0.23^***^	0.20^***^	−0.09^*^
2. Fear for one's family—COVID-19 (1–5)	3.46 (1.12)		0.36^***^	0.43^***^	0.34^***^	0.24^***^	0.25^***^	−0.04
3. Fear for one's self—terrorism (1–5)	1.94 (1.09)			0.80^***^	0.32^***^	0.16^***^	0.17^***^	−0.06
4. Fear for one's family—terrorism (1–5)	2.36 (1.28)				0.31^***^	0.07	0.15^***^	−0.04
5. Financial concerns (1–5)	2.74 (1.24)					0.19^***^	0.24^***^	−0.07
6. Coping: problem-focused (0–3)	1.33 (0.56)						0.55^***^	0.14^***^
7. Coping: emotion-focused (0–3)	1.01 (0.37)							−0.01
8. Resilience: total (0–100)	67.58 (13.12)							

* *p < 0.05*,

** *p < 0.01*,

*** *p < 0.001*.

Of the demographic variables, age was negatively associated with fear of COVID-19 with regard to one's family (*r* = –0.24, *p* < 0.001), fear of terrorism with regard to oneself (*r* = −0.12, *p* = 0.008), and fear of terrorism with regard to one's family (*r* = −0.13, *p* = 0.002). Overall, fear was higher among participants whose education level was lower than academic [fear of COVID-19 with regard to oneself: *M* = 2.87, *SD* = 1.25 vs. *M* = 2.49, *SD* = 1.05, *t*(375.56) = 3.77, *p* < 0.001; fear of COVID-19 with regard to one's family: *M* = 3.61, *SD* = 1.15 vs. *M* = 3.38, *SD* = 1.10, *t*(612) = 2.48, *p* = 0.013; fear of terrorism with regard to oneself: *M* = 2.09, *SD* = 1.25 vs. *M* = 1.86, *SD* = 0.98, *t*(355.63) = 2.39, *p* = 0.017; and fear of terrorism with regard to one's family: *M* = 2.59, *SD* = 1.37 vs. *M* = 2.24, *SD* = 1.21, *t*(389.90) = 3.10, *p* = 0.002].

Thus, the first step in the regression models included area (1-conflict area, 0-central Israel), gender (1-male, 0-female), age, education level (1-academic, 0-non-academic), and financial concerns. Coping strategies and resilience were entered in the second step. The results are presented in Table 4.

**Table 4:** Multiple hierarchical regressions for fear of COVID-19 and terrorism (*N* = 615).

	**Fear of COVID-19 with regard to oneself**		**Fear of COVID-19 with regard to one's family**		**Fear of terrorism with regard to oneself**		**Fear of terrorism with regard to one's family**	
	***B* (*SE*)**	**β**	***B* (*SE*)**	**β**	***B* (*SE*)**	**β**	***B* (*SE*)**	**β**
**Step 1**								
Gender	−0.04(0.09)	−0.02	−0.13(0.09)	−0.06	−0.44(0.08)	−0.22^***^	−0.25(0.10)	−0.12^*^
Area	−0.08(0.09)	−0.04	0.05(0.09)	0.02	0.10(0.08)	0.05	0.26(0.10)	0.13^**^
Age	0.01(0.01)	0.03	−0.02(0.01)	−0.20^***^	−0.01(0.01)	−0.08	−0.01(0.01)	−0.13^**^
Education	−0.28(0.09)	−0.12^**^	−0.20(0.09)	−0.08^*^	−0.22(0.09)	−0.11^*^	−0.28(0.11)	−0.13^**^
Financial concerns	0.33(0.03)	0.36^***^	0.27(0.03)	0.30^***^	0.25(0.03)	0.31^***^	0.29(0.04)	0.36^***^
Adj.*R*^2^	0.18^***^		0.17^***^		0.16^***^		0.14^***^	
**Step 2**								
Gender	0.10(0.09)	0.04	−0.01(0.09)	−0.01	−0.39(0.09)	−0.19^***^	−0.22(0.10)	−0.11^*^
Area	−0.05(0.09)	−0.02	0.08(0.09)	0.03	0.11(0.08)	0.06	0.27(0.10)	0.13^**^
Age	0.01(0.01)	0.04	−0.01(0.01)	−0.18^***^	−0.01(0.01)	−0.07	−0.01(0.01)	−0.13^*^
Education	−0.34(0.09)	−0.14^***^	−0.25(0.09)	−0.11^**^	−0.24(0.09)	−0.12^**^	−0.26(0.11)	−0.13^*^
Financial concerns	0.28(0.03)	0.31^***^	0.23(0.03)	0.26^***^	0.23(0.03)	0.28^***^	0.28(0.04)	0.35^***^
Coping: problem-focused	0.42(0.09)	0.21^***^	0.29(0.09)	0.15^**^	0.14(0.09)	0.08	−0.07(0.11)	−0.04
Coping: emotion-focused	0.10(0.14)	0.03	0.27(0.14)	0.09^*^	0.07(0.14)	0.03	0.25(0.16)	0.09
Resilience	−0.01(0.01)	−0.09^*^	−0.01(0.01)	−0.04	−0.01(0.01)	−0.03	−0.01(0.01)	−0.02
Adj.*R*^2^	0.22^***^		0.21^***^		0.17^***^		0.14^***^	

* *p < 0.05*,

** *p < 0.01*,

*** *p < 0.001*.

Results show that all four models are significant, explaining 14–22% of the variance in fear of COVID-19 and terrorism. Lower levels of education and higher levels of financial concerns were associated with higher levels of all types of fear. In addition to these variables, relationships were different by type of fear.

Fear of COVID-19 with regard to oneself was higher with greater use of problem-focused coping and lower resilience, in addition to lower education and higher financial concerns. Fear of COVID-19 with regard to one's family was higher with younger age, lower education, and higher financial concerns, as well as with greater use of problem- and emotion-focused coping. Fear of terrorism with regard to oneself was higher among women than men, and higher with lower education and greater financial concerns. It was unrelated to coping strategies and resilience. Fear of terrorism with regard to one's family was higher among females than males, higher in conflict areas, higher with younger age, and higher with lower education and greater financial concerns. It was unrelated to coping strategies and resilience.

## Discussion

The aim of the study was to explore whether living under constant security threat will result in better coping and higher resilience when exposed to an unknown threat such as COVID-19. Thus, we examined differences between fear of COVID-19 and fear of terrorism as well as associations with coping strategies and with resilience among those living in conflict zones compared to those living in the center, where exposure to security incidents is rare.

Contrary to our assumption, those who were living in a conflict zone did not exhibit lower levels of fear. Fear of COVID-19 was found to be much higher than fear of terrorism among both groups. In addition, those who were living in a conflict zone did not exhibit higher levels of resilience and did not cope better when exposed to a new threat—even though they may be more skilled at handling prolonged exposure to a threat such as terrorism. It seems that living under a continuous uncontrollable threat did not translate into enhanced ability to handle other life threats, nor did it lower that ability.

A possible explanation may be related to the nature of the new threat. Fear of COVID-19 and fear of terrorist attacks have similar roots. Both these events threaten to tear down the social fabric of life by creating a sense of fear and interfering with normal daily life routines (Cohen-Louck and Levy, 2021). However, whereas terrorism and security incidents are relatively well-known threats, COVID-19 poses a new type of stressor for Israeli society. The pandemic threat posed a new situation for which people cannot rely on their previous experience.

We assume that the findings are affected by the period during which the study was conducted. As mentioned, the data was collected at the beginning of the pandemic, during the peak of the first COVID-19 lockdown in Israel, when uncertainty and lack of knowledge concerning the virus and its effects were at their height. In times of public danger such as natural disasters and health emergencies, access to up to date information makes individuals and groups more resilient and less worried (Longstaff and Yang, 2008). Previous findings on public and individual fears indicates that perception of fear depends not only on the gravity of being a victim, but also on people's subjective perception of the likelihood of being a victim and of controlling whether they will be victimized (Warr, 1987; Jackson, 2011). Being in an uncerta*in situ*ation may explain why higher levels of COVID-19 fear were found among Israeli citizens, although none of the participants had contracted the virus and only 8 participants (1.3%) reported having had direct contact with someone who had become infected with COVID-19 at the time of collecting the data.

Other possible explanation may be related to the finding raised by the regression analysis. The best predictor of both fear of COVID-19 and of terrorism is worry due to the financial situation, beyond geographic area. In Israel, the most significant effect of the coronavirus was the need to stop working and to remain at home during the lockdown, with no knowledge of when and even whether workers would return to their jobs. At least in the first stage of the pandemic, most government efforts were directed at preventing the pandemic from spreading and less attention was given to COVID-19's financial implications (Shechory Bitton and Laufer, 2021). A similar situation was found in other countries, indicating that worrying about unemployment and financial loss were found to be associated with psychological maladjustment (Guo et al., 2020; Song et al., 2020; Trzebiński et al., 2020). Endangering economic stability was also found to be a salient aspect of terrorism. Economic loss and financial worry were found to be a major predictor of increased trauma-related symptoms following continuous exposure to security events (e.g., Stein et al., 2018). It seems that financial fear is a source of distress, which lowers the overall ability to confront other stressors such as the pandemic and terrorism.

These findings can be explained by the Conservation of Resources (COR) theory (Hobfoll, 1989), conceptualized as a bridge between environmental and cognitive viewpoints of adaptation to stress. The COR theory predicts that resource loss is a principal component of the stress process. According to Hobfoll (2001), environmental circumstances often threaten or generate a depletion of people's resources, threatening their status, position, economic stability, loved ones, etc. Consequently, loss of many resources due to trauma or crisis impairs the individual's adaptive abilities (Hobfoll and Lilly, 1993).

An interesting finding may support the significant impact of economic concerns on the well-being of those exposed to prolong stressful situations. A small significant effect was found for geographical area, with participants from conflict areas showing higher fear overall than participants from central Israel. However, These findings should be addressed, as they are surprising and are incongruent with former studies showing a habituation process to an ongoing threat among similar populations exposed to ongoing security and terrorism events (e.g., missile attacks) (e.g., Shechory Bitton and Laufer, 2017; Shechory Bitton and Silawi, 2019).

The second hypothesis was partially supported by the findings. Resilience were negatively associated with higher levels of fear of COVID-19. In addition, use of problem-focused coping and of emotion-focused coping were both positively associated with higher levels of fear of COVID-19. However, fear of terrorism was unrelated to coping strategies and resilience. In the current study, participants were asked to rate the extent to which they used each coping option to deal with stressful situations such as exposure to COVID-19. Thus, we believe that in their responses—they addressed the threat of the new threat.

This result can be explained by the “coping-panic cycle” hypothesis in which the more coping there is (whether emotion-focused or problem-focused), the more pandemic-related fear and distress (Huang et al., 2020; Man et al., 2020). According to this hypothesis, higher use of different forms of coping is mainly a manifestation of elevated stress and distress in the context of COVID-19 which, as a pandemic, is both a new and a relatively uncontrollable threat. That is, the more one is distressed, the more he or she will use different types of coping.

Several theorists (e.g., Lazarus and Folkman, 1984; Zeidner and Saklofske, 1996) highlighted the importance of matching the coping effort with the controllability of the situation. Consequently, when the personal risk is perceived to be high, the coping ability may be undermined, thus affecting the overall levels of fear (Tzur Bitan et al., 2020) and the individual's perceived coping potential, as well as the psychological resources needed to overcome a potential threat (Taylor and Stanton, 2007). The COVID-19 pandemic certainly fits the definition of an event perceived as uncontrollable or lacking a viable solution (Shechory Bitton and Laufer, 2021). It is logical for participants to use emotional (e.g., concerns about health, especially for one's family and one's financial situation) in conjunction with practical coping strategies (e.g., attempts to protect themselves as well as their family). As mentioned before, the beginning of the pandemic was characterized by a general sense of confusion, resulting from the dramatic changes required to cope with the virus (Reizer et al., 2020). At that point, the media was saturated with information describing individual hardships, the extremely high infection rate, and the relatively high mortality. Presumably, this is why coping strategies in the present study were associated with a higher level of fear.

The same argument may also apply for explaining the association between lower resilience and higher levels of fear for oneself due to COVID-19. Resilience defined as the ability to cope successfully with stressful and traumatic events (Bonanno, 2004; Straud et al., 2018), an ability that has been associated with coping strategies (Reich et al., 2010). Growing research continues to find evidence in support of the notion that positive emotions have the ability to widen the range of potential coping strategies during times of stress, consequently enhancing one's resilience against present and future adversity, and vice a versa (e.g., Gloria and Steinhardt, 2016). Resilience as a personal characteristic embodies the personal qualities as well as an individual's past life experiences and current life circumstances enabling one to thrive in the face of adversity. In the present study, resilience was examined as a personality trait (Connor and Davidson, 2003). Hence, it is possible that this type of resilience is more relevant for stressors that threaten the self.

Finally, in line with previous findings (e.g., Laufer et al., 2019; Qiu et al., 2020; Wang et al., 2020; Shechory Bitton and Cohen-Louck, 2021), women were found to display more fear than men. They also used more coping strategies and had less resilience compared to men. These findings are in line with other findings (for review see: Tamres et al., 2002; Hu et al., 2015). Hence, the tendency of women to be more threatened by a stressor, especially a life threatening unmanageable one such as terror and pandemic, is consistent. It may be that, as the panic-coping hypothesis posits, women use more coping techniques since they feel more stressed.

Overall, the results of this study indicate that individuals who are accustomed to reacting to continuous uncontrollable life threats such as terror and missiles are not more capable of managing other life threatening stressors and they are at risk of being overwhelmed by a new stressor. These results need further examination regarding differences and similarities between stressors and reactions to stressors in order to enhance our understanding of the ability to “generalize” from one experience to another. An unanswered question resulting from the current study is whether a process of habituation will emerge in time following exposure to COVID-19.

Perhaps precisely since the study was conducted at the very beginning of the pandemic, when uncertainty was very high, and although the participants were not directly affected by the virus, the reaction was similar to that found in other one-time or short time incidents, even without direct (objective) exposure. For instance, in studies conducted after 9/11, high levels of fear and distress (subjective exposure) were found, unrelated to people's objective exposure (Bonanno et al., 2006). At the time these lines are being written, many months after the study was conducted, the COVID-19 pandemic has not yet ended. Prolonged exposure to situations of tension and stress require those exposed to find practical solutions, despite feelings of fear (Shechory Bitton and Laufer, 2017). There is room for further studies that will address the ramifications of the prolonged exposure to the COVID-19 pandemic and check whether a process of habituation occurred over time, which moderated the fear levels. Integrating some other core aspects into existing explanations could help uncover some of the dynamics and mechanisms underpinning important current day phenomena.

Some potential limitations should be noted. We relied on convenience sampling by using an online survey. This may limit the study's ability to reach all strata of the Israeli population. In addition, a cross-sectional design does not allow for causal inferences. Additional longitudinal studies, such as cohort studies or nested case-control studies, are essential for future research (e.g., Gao et al., 2020). Furthermore, self-report measures were used, and some had a single item. Finally, no measures of the consequences of both fears were included in the current study, and these are recommended for future analyses. It should also be noted that in the current study fear is a measure of distress resulting from the situation, however when dealing with terrorism or a new virus fear may be a sign of adjustment to the situation, helping one to sustain and survive.

Despite its limitations, this research has a novel contribution and entails several important implications. One of the paper's strengths is that it addresses two issues that are relevant and significant for extensive parts of the world (not only Israel): dealing with terrorism and dealing with the COVID-19 crisis. The study was conducted at the beginning of the crisis (when there was a high sense of uncertainty, and accordingly of fear). As far as known, this is the first empirical study to explore whether experience with continuous exposure to stressful situations (security threats and terrorism) can help cope with a new exceptional source of stress (“invisible enemy”). The findings can help understand processes of resilience and of coping with stressful situations. The findings show that fear is not simply a measure of the outcome of exposure to stress or a threatening situation. Identifying levels of fear among different populations and especially their relationship to specific sociodemographic variables such as geographical location, level of education, financial situation, and gender, could assist in locating potential risk groups.

## Data Availability Statement

The raw data supporting the conclusions of this article will be made available by the authors, without undue reservation.

## Ethics Statement

The studies involving human participants were reviewed and approved according to the ethical standards of the Institutional Review Board (IRB) of Ariel University. The patients/participants provided their written informed consent to participate in this study.

## Author Contributions

MS and AL contributed to conception and design of the study, manuscript writing, and approved the submitted version. MS led questionnaire development and organized the database. All authors contributed to the article and approved the submitted version.

## Conflict of Interest

The authors declare that the research was conducted in the absence of any commercial or financial relationships that could be construed as a potential conflict of interest.
